# Syndecan-1 Predicts Outcome in Patients with ST-Segment Elevation Infarction Independent from Infarct-related Myocardial Injury

**DOI:** 10.1038/s41598-019-54937-x

**Published:** 2019-12-04

**Authors:** Bernhard Wernly, Georg Fuernau, Maryna Masyuk, Johanna Maria Muessig, Susanne Pfeiler, Raphael Romano Bruno, Steffen Desch, Phillip Muench, Michael Lichtenauer, Malte Kelm, Volker Adams, Holger Thiele, Ingo Eitel, Christian Jung

**Affiliations:** 1 Department of Internal Medicine II, Division of Cardiology, Paracelsus Medical University, Salzburg, Austria; 2 University Heart Center Lübeck, Medical Clinic II, University Hospital Schleswig-Holstein and German Center for Cardiovascular Research (DZHK), Partner Site Hamburg/Kiel/Lübeck, Lübeck, Germany; 3 Division of Cardiology, Pulmonology, and Vascular Medicine, Medical Faculty, University Duesseldorf, Duesseldorf, Germany; 4 Department of Internal Medicine/Cardiology, Heart Center Leipzig at University of Leipzig and Leipzig Heart Institute, Leipzig, Germany; 5 Laboratory of Molecular and Experimental Cardiology, Heart Centre Dresden, TU Dresden, Dresden, Germany

**Keywords:** Interventional cardiology, Myocardial infarction

## Abstract

Syndecan-1 (sdc1) is a surface protein part of the endothelial glycocalyx (eGC). Soluble sdc1 is derived from shedding and indicates damaged eGC. We assessed the predictive value of plasma sdc1 concentrations for future cardiovascular events in acute reperfused ST-segment elevation myocardial infarction (STEMI) patients. A total of 206 patients admitted for STEMI were included in this study (29% female; age 65 ± 12 years) and followed-up for six months. Plasma samples were obtained post-intervention and analyzed for sdc1 by Enzyme-linked Immunosorbent Assay (ELISA). Primary outcome was six-month-mortality. Sdc1 did not correlate with biomarkers such as creatine kinase (CK) (r = 0.11; p = 0.01) or troponin (r = −0.12; p = 0.09), nor with infarct size (r = −0.04; p = 0.67) and myocardial salvage index (r = 0.11; p = 0.17). Sdc-1 was associated with mortality (changes per 100 ng/mL sdc-1 concentration; HR 1.08 95% 1.03–1.12; p = 0.001). An optimal cut-off was calculated at >120 ng/mL. After correction for known risk factors sdc1 >120 ng/mL was independently associated with mortality after 6 months. In our study, sdc1 is independently associated with six-month-mortality after STEMI. Combining clinical evaluation and different biomarkers assessing both infarct-related myocardial injury and systemic stress response might improve the accuracy of predicting clinical prognosis in STEMI patients.

## Introduction

In acute ST-elevation myocardial infarction (STEMI) reperfusion therapy has improved outcomes dramatically. However, STEMI is still accompanied by high mortality and morbidity. After initial therapy, consisting of primary percutaneous coronary intervention (PCI) and treatment of acute complications, optimal medical treatment, and cardiac rehabilitation improve outcomes^1,2^. Due to limited resources in health care, it is essential to identify patients at very high risk for adverse outcomes to monitor these patients closer and to treat complications and secondary risk factors early and aggressively.

Several scoring systems have been developed and established in STEMI patients, with Global Registry of Acute Coronary Events (GRACE) score, Killip and Thrombolysis In Myocardial Infarction (TIMI) scores being the most widely used^3,4^. Others investigated micro-RNAs or established imaging markers especially by cardiac magnetic resonance (CMR) as new tools for the prediction of future cardiovascular events after STEMI^5–7^. However, CMR is difficult to obtain in daily clinical practice in all STEMI patients because of limited availability^8^ and micro-RNAs are very expensive and time-consuming to measure. Therefore, new tools for risk prediction after STEMI are warranted.

Syndecan-1 (sdc1) is a surface protein on endothelial cells^9^. Surface proteins, also termed endothelial glycocalyx (eGC), maintain the barrier between blood and endothelium, preventing extravasation of water, proteins and electrolytes^10,11^. Oxidized low-density lipoprotein cholesterol was shown to degrade eGC linking dyslipidemia with atherosclerosis^9^. Serum soluble sdc1, indicating impaired eGC, was shown to correlate with catecholamine levels in patients suffering from STEMI^12^ and for patients with cardiogenic shock complicating acute MI an association to mortality has been shown recently^13^. Further, in patients with chronic heart failure, high sdc1 was associated with higher mortality at six months^14^. We therefore assessed sdc1 for risk prediction of mortality and major adverse cardiac events in STEMI patients following reperfusion by primary PCI^15^.

## Methods

### Study population and clinical endpoint

This is a predefined biomarker sub-study of the LIPSIA CONDITIONING (Effect of Conditioning on Myocardial Damage in STEMI) trial, an open-label, randomized controlled trial conducted at the University of Leipzig-Heart Center between April 2011 and May 2014 (ClinicalTrials.gov Identifier: NCT02158468)^15^. Design and results of the study have been published, and parts of this study cohort were investigated and published in another context^15–18^. Exclusion criteria in the original study were cardiogenic shock, limited life expectancy below six months, age below 18 years, pregnancy, previous fibrinolysis, contraindications to CMR imaging and participation in another trial.

In this sub-study, 206 patients of the control arm were included. Primary endpoint of this study was mortality within six months, and a combined clinical endpoint of major adverse cardiac events (MACE) consisting of death, re-infarction, and development of congestive heart failure within six months was defined as a secondary endpoint. One patient was lost to follow-up. The study was approved by the local ethics committee of the University of Leipzig-Heart Center, and all patients provided written informed consent. All methods were performed in accordance with the relevant guidelines and regulations.

### Healthy controls

In total, plasma samples from 20 healthy controls (10 male, 29 ± 4 years, no comorbidities) were collected.

### Cardiac magnetic resonance imaging

Detailed information about CMR imaging protocols were published previously^15–17^. In short, CMR was performed between day two and five after STEMI using a 1.5-T scanner. Myocardial salvage, infarct size, microvascular obstruction, left ventricular volumes and ejection fraction were calculated. Blinded readers evaluated images at a core laboratory with vast experience in CMR trials^7^.

### Determination of syndecan

Plasma samples for detection of sdc1 were obtained after PCI for STEMI. Plasma levels of sdc1 were determined using enzyme-linked immunosorbent assay (ELISA, Diaclone, France) according to the manufacturer’s protocol. In short, plasma samples and standard protein were added to the wells and incubated for two hours. After this incubation period plates were washed three times. Then, a biotin-labeled secondary antibody was added, and the plates were incubated for another two hours. Later, the plates were washed again, and Streptavidin-horseradish-peroxidase was added. Color reaction was achieved using tetramethylbenzidine (TMB; Sigma Aldrich, USA). Optical density values were measured at 450 nm on an ELISA plate-reader (Bio-Rad Laboratories, Austria).

### Statistical analysis

Statistical analysis was performed using SPSS (23.0, SPSS Inc., USA) and MedCalc Statistical Software version 18.11.3 (MedCalc Software, Ostend, Belgium). Spearman correlation analysis was used to analyze the association of sdc1 concentration with CMR measurements and other biomarkers. Continuous data are given as mean and standard deviation (SD) and compared by ANOVA.

Receiver operating curve (ROC) analysis was performed, area under the curve (AUC) calculated and an optimal cut-off calculated using the Youden-Index. Patients were retrospectively divided into two cohorts: those above the optimal cut-off and those below. Survival was depicted using Kaplan-Meier method and log-rank-testing.

Univariable Cox, proportional hazards analysis, was used to identify factors - after reviewing of available literature - associated with an increased risk of mortality at six months and hazard ratios (HR) with 95% confidence intervals (CI) were obtained. For a multivariable Cox regression model, confounders with a p-value < 0.10 in the univariate analysis were included, then a backward variable elimination was performed. Elimination criterion was a p-value of more than 0.10. Known risk factors such as concomitant diseases (diabetes, hyperlipidemia, hypertension) were forced into the model.

An integrative score was developed after a review of the literature and with the aim of being 1) of high discriminative power and 2) easy to calculate. The integrative score was developed as described elsewhere^19,20^. Killip class was included as an ordinal variable. For age, troponin and sdc1 an optimal cut-off for prediction of mortality after six months was calculated by means of the Youden index. All variables were included in logistic regression and regression coefficients (β) obtained. Age, troponin and sdc1 were dichotomized in above and below their respective optimal cut-off, for Killip class I was set as the reference category. Based on these β, distances to the reference values were calculated and the number of points for each category was determined. Then the integrative score was calculated for each individual patient.

Categorial variables were compared by chi-square test. A p-value of <0.05 was considered statistically significant.

### Ethics approval and consent to participate

A study protocol was provided to participating centers. Every participating center obtained ethics approval according to local legislation. A copy of the ethics approval was sent to the study coordinator before start of the study.

### Consent for Publication

Written informed consent was obtained of all included subjects, unless the local ethics committee specifically allowed a waiver in this respect. The study was registered at http://www.clinicaltrials.gov/ (NCT02731898).

## Results

Median sdc1 levels in the healthy controls were 11 ng/mL (range 5–47 ng/mL). In the 206 patients sdc1 was measured, and median sdc1 was 62 ng/mL (p < 0.0001 vs healthy controls). Sdc1 was associated with mortality in a univariable Cox regression analysis (changes per 100 ng/ml sdc1 concentration; HR 1.08 95%CI 1.03–1.12); p = 0.001). AUC for prediction of mortality was 0.76; 95%CI 0.69–0.81, and an optimal cut-off was calculated using the Youden index at >120 ng/mL. Scd1 was also associated with MACE (HR 1.06; 95%CI 1.02–1.10; p = 0.001).

Patients with sdc1 concentration >120 ng/mL (n = 147) were older (68 ± 10 vs 63 ± 2 years; p = 0.003) and more often male (76% vs 61%; p = 0.04) compared to patients with lower sdc1 (Table 1). Concomitant diseases such as diabetes, hypertension and hyperlipidemia were not significantly different. There was a non-significant trend towards higher rates of Killip class III and IV (3% vs 12%; p = 0.053) in the sdc1 >120 ng/mL group. Of note, in patients with Killip class >I, sdc1 (149.37 ± 384.48 versus 240.07 ± 314.27; p = 0.28) was not dissimilar to patients in Killip I class. Discharge medication was similar between patients with sdc1 concentration >120 ng/mL vs ≤120 ng/mL (Table 1). Traditional biomarkers such as maximum troponin (3711 ± 3020 versus 3527 ± 2839; p = 0.68) and maximum creatine kinase (CK) levels (39 ± 53 versus 29 ± 27; p = 0.08) were not significantly different between patients with sdc1 concentration >120 ng/mL and ≤120 ng/mL (Table 2). The same holds true for procedural aspects including rates of thrombectomy and direct stenting (Table 2).

**Table 1:** Baseline characteristics, concomitant diseases and medication at discharge of patients with sdc1 >120 vs. ≤120 ng/ml; continuous data in mean ± SD; categorial data in percentage.

	Sdc1 ≤120 ng/mL		Sdc1 >120 ng/mL		total cohort		p-value
	n = 147		n = 59		n = 206		
age (years)	62.99	12.15	68.29	10.42	64.51	11.90	0.003
BMI	27.79	3.92	27.82	4.61	27.80	4.12	0.96
Symptom onset to PCI hospital admission (min)	246.04	184.89	260.03	156.75	250.09	176.94	0.61
Door-To-Balloon-Time (min)	28.85	12.71	38.31	58.93	31.54	33.30	0.07
male sex	76%		61%		71%		0.04
Current smoking	39%		44%		41%		0.64
Hypertension	78%		69%		75%		0.28
Hypercholesterinemia	43%		49%		45%		0.44
Diabetes mellitus	24%		27%		25%		0.72
Previous infarction	9%		12%		10%		0.60
Previous PCI	9%		7%		8%		0.78
**Haemodynamics**							
Killip class on admission							0.052
I	90%		85%		89%		
II	6%		3%		5%		
III or IV	3%		12%		6%		
IABP use	1%		7%		2%		0.02
**Concomitant medications at discharge**							
Aspirin	99%		98%		99%		1.00
Clopidogrel	31%		37%		33%		0.42
Prasugrel	68%		63%		67%		0.52
Ticagrelor	15%		17%		16%		0.83
beta-blockers	97%		98%		98%		1.00
ACE-I/ARB	97%		97%		97%		1.00
Statins	97%		92%		95%		0.15

BMI = body mass index; PCI = percutaneous coronary intervention; IABP = intra-aortic balloon pump, ACE-I = angiotensin converting enzyme inhibitor; ARB = angiotensin II receptor blocker.

**Table 2:** Biomarker, angiographic and procedural characteristics of patients with sdc1 >120 vs. ≤120 ng/ml; continuous data in mean ± SD; categorial data in percentage.

	Sdc1 ≤120 ng/mL		Sdc1 >120 ng/mL		total cohort		p-value
	n = 147		n = 59		n = 206		
**Biomarkers**							
Syndecan (ng/mL)	42.25	36.13	451.63	615.86	159.50	377.70	0.00
CK-max (U/L)	29.20	26.72	39.00	53.27	32.01	36.48	0.08
CK-MB max (U/L)	2.96	2.18	3.61	2.98	3.14	2.44	0.09
Troponin (ng/L)	3526.49	2838.94	3710.71	3019.61	3578.87	2885.13	0.68
Creatinine	85.05	27.92	107.27	107.36	91.41	62.59	0.02
Anterior infarction	52%		47%		50%		0.65
TIMI flow before PCI							0.40
0	55%		51%		54%		
I	10%		15%		11%		
II	19%		24%		20%		
III	16%		10%		15%		
TIMI flow after PCI							0.28
0	3%		0%		2%		
I	3%		0%		2%		
II	10%		10%		10%		
III	84%		90%		85%		
Stent implanted	95%		97%		96%		0.81
Direct stenting	78%		76%		77%		0.85
Thrombectomy	62%		61%		62%		1.00

CK = creatine kinase; CK-MB = creatine kinase-muscle/brain; TIMI = Thrombolysis In Myocardial Infarction; PCI = percutaneous coronary intervention

CMR imaging revealed no significant differences in left ventricular (LV) ejection fraction, infarct size, myocardial salvage or microvascular obstruction between patients with sdc1 >120 ng/mL vs ≤120 ng/mL (Table 3). Sdc1 did not correlate with biomarkers such as CK (r = 0.11; p = 0.011) or troponin (r = −0.12; p = 0.09), nor with infarct size (r = −0.04; p = 0.67) and myocardial salvage index (r = 0.11; p = 0.17) assessed by CMR.

**Table 3:** CMR characteristics of patients with sdc1 >120 vs. ≤120 ng/ml; continuous data in mean ± SD; categorial data in percentage.

	Sdc1 ≤120 ng/mL		Sdc1 >120 ng/mL		total cohort		p-value
	n = 146		n = 59		n = 205		
LV ejection fraction (%)	48.89	12.35	46.76	12.62	48.33	12.42	0.34
LV end-diastolic volume (mL)	143.61	44.52	139.29	45.65	142.50	44.72	0.59
LV end-systolic volume (mL)	76.21	39.11	76.83	39.61	76.37	39.12	0.93
Infarct size (%LV)	19.54	14.17	19.45	13.02	19.51	13.83	0.97
microvascular obstruction (%LV)	1.69	3.50	1.41	2.65	1.62	3.29	0.64
Myocardial salvage (%LV)	13.22	9.95	15.64	9.67	13.84	9.90	0.20
Myocardial salvage index	42.86	28.18	47.96	29.16	44.18	28.42	0.34

**Figure 1:**
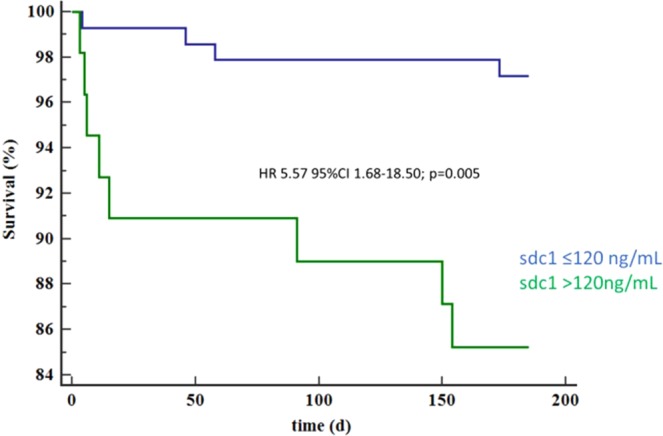
Survival at 6 months of patients with sdc1 >120 vs. ≤120 ng/ml.

**Figure 2:**
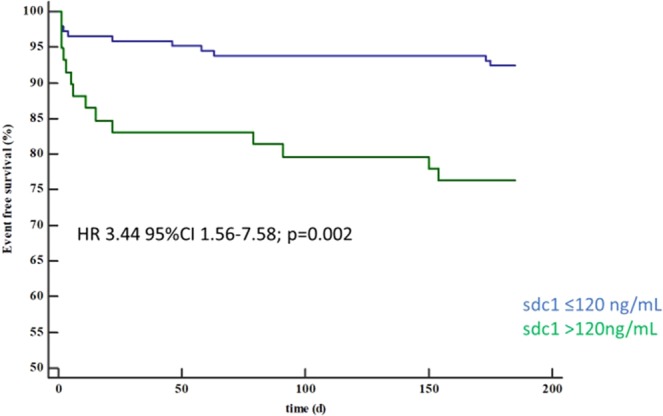
MACE-free survival at 6 months of patients with sdc1 >120 vs. ≤120 ng/ml.

Patients with sdc1 >120 ng/mL evidenced higher mortality (HR 5.57; 95%CI 1.68–18.50; p = 0.005; Fig. 1) after six months. Further, patients with sdc1 >120 ng/mL showed also higher rates of MACE (HR 3.44; 95%CI 1.56–7.58; p = 0.002; Fig. 2) after six months. This was mainly driven by mortality (8 of 59 patients [14%] vs 4 of 146 [3%]; p = 0.006), whereas rates of reinfarction (1 of 59 [2%] vs 0 of 146 [0%]; p = 0.29) and development of congestive heart failure (5 of 59 [9%] vs 7 of 146 [5%]; p = 0.29) were low and not significantly different.

The association of sdc1 >120 ng/mL with MACE remained significant after correction for known risk factors for cardiovascular disease (male sex, diabetes, hypertension, age, hyperlipidemia, smoking; HR 3.31; 95%CI 1.48–7.41; p = 0.004) and established biomarkers such as maximum CK (HR 3.21; 95%CI 1.43–7.18; p = 0.005) as well as troponin at presentation (HR 3.15; 95%CI 1.35–7.32; p = 0.008). Further, sdc1 >120 ng/mL remained associated with MACE after six months even after correction for Killip class >I (HR 3.13; 95% CI 1.41–6.92; p = 0.005).

Compared to other biomarkers such as troponin (AUC 0.68; 95% CI 0.62–0.75; p = 0.74 vs. sdc1) and CK (AUC 0.60; 95% CI 0.53–0.67; p = 0.35 vs. sdc1) sdc1 had numerically higher discriminative power (AUC 0.76; 95%CI 0.69–0.81). We combined troponin, sdc1, age and Killip class in an integrative score as described above to test the concept of combining distinct biomarkers and clinical assessment and calculated this score for each patient. The integrative score had high discriminative power (AUC 0.96; 95%CI 0.92–0.98) and was significantly associated with mortality (per score point; HR 1.86; 95%CI 1.49–2.31; p < 0.001).

## Discussion

Compared to healthy individuals, scd1 concentration were significantly higher in STEMI patients. To the best of our knowledge this study is the first to demonstrate an independent relationship of plasma sdc1 levels with mortality and MACE six months after STEMI. Increased neointimal hyperplasia and a reduction of macrophage motility in animal models of sdc1-null-mice suggest a role of sdc1 in arterial healing processes^21,22^. In a mouse myocardial infarction model increased sdc1 expression reduced cardiac dilatation and dysfunction^23^. An intact eGC is known to decrease coagulation via leucocyte and platelet adhesion^9,24^. Contrary, soluble eGC components with sdc1 being the most abundant thought to stem from shedding are associated with an inflammatory state and immunosuppression^25^. Further, eGC was shown to predict one-month mortality in critically ill patients suffering from cardiogenic shock due to myocardial infarction^13^. In our study of STEMI patients soluble sdc1 was a strong and independent predictor of mortality, even after correction for relevant confounders, as long as six months after STEMI. This contrasts data from Vanhoutte *et al*. who could show that myocardial Sdc-1 expression attenuated cardiac inflammation and remodeling after myocardial infarction^23^. However, others reported an inverse association of glycocalyx shedding assessed by Sdc-1 plasma levels and LV function in humans^26^.

The exact physiological and pathophysiological role of sdc1 in cardiac ischemia and ischemic heart failure are beyond the scope of this paper. One could, however, speculate that increased sdc1 shedding after STEMI leads to impaired eGC and thereby alters arterial healing processes and leads to adverse outcome^21,22^. Of note, Sdc-1 concentrations might not only be influenced by glycocalyx shedding: Tromp *et al*., reported associations of Sdc-1 with fibrosis in chronic heart failure patients^27^. However, Sdc-1 correlated with levels catecholamines in STEMI patients^12^. Further, in STEMI patients with hemodynamic impairment, sdc-1 levels were higher compared to hemodynamically stable patients^28^. Therefore, in patients with STEMI, sdc-1 concentrations could primarily reflect the acute pathophysiology rather than long-term fibrotic or remodeling changes.

Interestingly, in our study soluble sdc1 levels did neither correlate with known markers of myocardial injury such as biomarkers nor with infarct size assessed by CMR^29^. Therefore, an increase in sdc1 might indicate an injury to eGC which is not directly linked to myocardial tissue damage including myocardium at risk, the extent of final myocardial necrosis and microvascular injury.

Further, patients in the sdc1 >120 ng/mL sub-group of this study evidenced similar baseline characteristics compared to patients below this cut-off regarding known cardiovascular risk factors. An sdc1 concentration above this cut-off remained predictive for outcome after correction for several potential confounders including sex and age. Sdc1 might represent a new tool to assess eGC injury after STEMI, and its evaluation might add another important piece to the puzzle of optimized risk stratification in STEMI patients. Certainly, the concept of sdc1 evaluating eGC injury after myocardial ischemia in STEMI is at this timepoint speculative. Further mechanistic studies evaluating sources of sdc1 in patients with STEMI are necessary. Of note, as sdc1 did not correlate with infarct size assessed by CMR but with clinical outcome, elevated sdc1 levels could indicate systemic eGC injury due to incipient cardiogenic shock. This would be in accordance with previous studies reporting sdc1 to be an independent predictor of one-month mortality in patients suffering from cardiogenic shock^13^. However, in the present study cohort, sdc1 did not correlate with Killip class.

Established scoring systems for risk prediction in acute coronary syndrome are relatively complex to calculate. Combining several biomarkers such as CK or troponin assessing myocardial tissue injury, and sdc1 assessing eGC injury, as well as simple clinical information such as congestion or blood pressure or lactate level, could be relatively easy to implement and still be very powerful in predicting risk of adverse outcome after STEMI^3,4,30^. For our study cohort, we developed a score consisting of Killip class, age, and both troponin and sdc1 concentrations: this integrative score evidenced high discriminative power and is relatively easy to calculate. The exclusion of patients in cardiogenic shock in this study might predispose this score to selection bias considering the inclusion of Killip class. Certainly, a score including these, and other parameters needs to be refined and validated in other larger cohorts including patients with cardiogenic shock and the high discriminative power in this cohort lacking independent validation might be data driven. Still, we think that the high discriminative power after combining clinical evaluation, age, a marker of myocardial injury – namely troponin – and a potential marker of eGC injury – namely sdc1 – in this small cohort at least supports the notion that this concept could add significant value to risk prediction models in STEMI and warrants further investigations.

### Limitations

The exact pathophysiological role of sdc1 and soluble sdc1 in myocardial injury in STEMI is beyond the scope of this paper, and therefore our conclusion remains thesis generating and warrants further research. Further, this pre-specified biomarkers sub-study of the LIPSIA CONDITIONING study focused on sdc-1 concentrations based on previous studies reporting associations of sdc-1 with hemodynamic alterations and outcomes in shock^12,13,28^. However, other syndecan proteins could be altered as well, and a comparison of distinct syndecan concentrations could bring further insight, but was beyond the scope of this study. This study was conducted only in a single center and the absolute patient number might be underpowered to assess the association of sdc1 and mortality in patients suffering from STEMI.

## Conclusion

In our study, we show that sdc1 is independently associated with mortality six months after STEMI. Sdc1 did not correlate with traditional markers of myocardial injury and might, therefore, constitute an independent tool for risk stratification, eventually by assessing eGC injury in STEMI patients. Combining clinical evaluation and biomarkers assessing different injuries might be powerful to predict risk in STEMI patients.

## Data Availability

All data relevant for this study will be given by the authors upon specific request without restriction.
